# Study of the Histopathologic Characteristics and Surface Morphologies of Glottic Carcinomas With Anterior Vocal Commissure Involvement

**DOI:** 10.1097/MD.0000000000001169

**Published:** 2015-07-24

**Authors:** Jianhui Wu, Jing Zhao, Zhangfeng Wang, Zenghong Li, Jie Luo, Bing Liao, Zhiyun Yang, Qihong Liu, Bin Wang, Weiping Wen, Wenbin Lei

**Affiliations:** From the Otorhinolaryngology Hospital (JW, JZ, ZW, QL, BW, WW, WL), The First Affiliated Hospital, Sun Yat-sen University, Guangzhou, Guangdong, China; Department of Surgery (ZL, JL), Queen Mary Hospital, The University of Hong Kong, Hong Kong; and Department of Pathology (BL, ZY), Department of Radiology, The First Affiliated Hospital, Sun Yat-sen University, 58 Zhongshan Road II, Guangzhou,Guangdong 510080, China.

## Abstract

This article explores the features and the role of the anterior vocal commissure (AVC) structure and the surface morphologies of glottic carcinomas with AVC involvement to provide a reference for the selection of transoral carbon dioxide (CO_2_) laser surgery.

A total of 31 cases of glottic carcinomas with AVC involvement from May 2012 to January 2014 were included. All patients underwent electronic laryngoscopic examinations and computed tomography scans to determine the surface morphology. After surgery, the tumor specimens were resected integrally, and axial serial sections parallel to the plane of vocal cords were taken to explore the features and possible invasion paths of the glottic carcinomas with AVC involvement.

The rates of involvement of the supraglottis and subglottis were 71.4% and 14.8%, respectively, via the AVC. The involvement of the superficial layer of the unilateral or bilateral vocal cords without involvement of the vocal muscle in the AVC region (IVM) or the cartilage was present in 15 cases (48.4%). The involvement of the superficial layer of the unilateral and bilateral vocal cords occurred in 16 cases (51.6%) with the IVM in 13 cases and the involvement of the intermediate lamina of the thyroid cartilage (ITC) in 8 cases. The involvement of the ITC was associated with the involvement of the vocal muscle of the AVC region (*P* < 0.05). Among the pushing carcinomas, 15 of 21 (71.4%) presented with well-defined tumor mass, and 8 of 10 (80.0%) infiltrating carcinomas presented with multiple tumor nests that were often surrounded by fibrosis (*P* < 0.05).

The AVC is an important path of invasion of subglottic in glottic carcinomas but less so for suparglottic. The Broyles’ ligaments acted as a barrier against the spread of the tumors to the thyroid cartilage, but this role was obviously weaken by the involvement of the vocal muscle of the AVC region. The infiltrating carcinomas presented with multiple tumor nests in fibrous tissue. When CO_2_ laser microsurgery is considered as a treatment option, these facts should be kept in mind.

## INTRODUCTION

Increasing evidence suggests that early glottic carcinomas can benefit from carbon dioxide (CO_2_) laser microsurgery because of some of the advantages of this treatment and the preservation of vocal function. However, a higher recurrence rate is observed in cases with anterior vocal commissure (AVC) involvement.^1,2^ The possible reasons for this observation may be correlated with insufficient AVC exposure and the invasive features of the tumors. Therapeutic strategies for early glottic carcinomas involving the AVC remain controversial.^3,4^

The histopathologic characteristics of regional tumor invasion and extension in the AVC remain uncertain. There are 2 special anatomic structures in the AVC region, the anterior macula flava and the Broyles’ ligament (the anterior commissure tendon), that contribute to vocal fold vibration and tumor invasion.^5,6^ Some studies have suggested that tumors that are originally located in the unilateral AVC can cross the midline to the contralateral part and also easily extend to the thyroid cartilage, and thus demonstrated that the AVC region is a weak point in tumor invasion.^7–9^ The reasons for this weakness include the following: the inner perichondrium is absent at the site at which Broyles’ ligament inserts into the thyroid cartilage, which makes the cartilage vulnerable to cancer invasion^10,11^; and because of the small distance (2–3 mm) between the AVC mucosa and the thyroid cartilage and the existence of multiple anatomical subregions of the larynx, even small tumors can reach and invade the cartilage and cause an instant change from an early stage tumor (T1) to an advanced laryngeal carcinoma (T3 or T4) and changes in therapeutic strategies.^12^ However, there are contradictory opinions about whether Broyles’ ligament can act as a barrier to prevent the tumoral invasion of the thyroid cartilage.^13^

In this study, we sought to explore the features and possible invasion paths of glottic carcinomas with AVC involvement based on histopathological serial sectioning and study the surface morphologies with electronic laryngoscopy and computed tomography (CT) examinations in an attempt to find evidence that is relevant to therapeutic strategy decisions.

## MATERIALS AND METHODS

### Ethics Statement

The research protocols were approved by the Ethics Committee of the First Affiliated Hospital of Sun Yat-sen University, Guangdong, China ([2013]160#). Written informed consent was obtained from each patient, and all patients granted us permission to use the data obtained in subsequent studies.

### Participants

This study was exclusively performed on cases of glottic carcinoma with AVC involvement. A total of 31 patients were recruited between May 2012 and January 2014 from the Department of Otolaryngology of the First Affiliated Hospital of Sun Yat-sen University. All of these patients were first discovered to have glottic squamous cell carcinomas with AVC involvement via electronic laryngoscopy examination, and the diagnoses were histologically confirmed with biopsies. None of the patients had accepted surgical resection, chemotherapy, or radiotherapy prior to admission.

### Clinical Data Collection

The tumor surface morphologies were determined by preoperative electronic laryngoscopy and enhanced CT examination (TOSHIBA Aquillion: 64 row, Aquillion, TOSHIBA company, Japan). According to the relation of the tumor and the surrounding normal tissues and the site of the main body of the tumor, we formed 2 groups: the pushing carcinomas (nodal, massive, or cauliflower types with clear tumor edges and main tumor bodies located in the vocal epithelial lamina or cavity), and the infiltrating carcinomas (deep ulcer type with unclear tumor edges and main tumor bodies located in the vocal propria lamina or the deeper layers).

All the patients underwent a transcervical operation. Total laryngectomy and frontal partial laryngectomy were performed on 8 and 10 patients, respectively, and 13 cases underwent cricoid partial laryngectomy.

### Specimen Processing

Regarding the specimens, at least 2 or 3 anterior vocal cords were retained, and the posterior parts were trimmed off. All of the samples were then cut into 3 to 6 segments (each segment was 6 to 8 mm thick) parallel to the plane of the vocal cords and subsequently fixed and decalcified. Next, the specimens were paraffin embedded in an upside–down position. Each paraffin-embedded segment was cut into axial serial slices (5 μm thick) parallel to the plane of vocal cords at 0.4-mm intervals for hematoxylin–eosin staining.

### Imaging and Histopathological Analysis

The tumor stages were determined according to the Union for International Cancer Control-2002 tumor node metastasis guidelines. Two senior radiologists were assigned to independently analyze the CT images, and 2 senior pathologists independently reviewed all of the serial sections with an optical microscope. The patients were classified according to the degree of the involvement of the AVC as follows^10^: patients without AVC region involvement (AVC0); patients with AVC region involvement on only one side of the midline (AVC1); patients with AVC region involvement that penetrated the midline on only part of the longitudinal extent of this region (AVC2); and patients in whom the entire AVC region was involved (AVC3). Both the imaging and histological results were obtained only when the 2 practitioners reached consensus opinions in a final discussion. The sections were scanned and saved with a digital slice scanner (Nanozoomer 2.0 HT; Hamamatsu Photonics, Hamamatsu, Japan).

### Statistical Analysis

Statistical analysis was performed with SPSS V13.0. The difference of accuracy was calculated using χ^2^ test. *P* value <0.05 was considered statistically significant.

## RESULTS

### Clinical Features of Participants

Thirty-one cases with glottic carcinomas with AVC involvement were included. The patients were men, and the median age was 60.0 ± 9.6 years (46–81 years; Table 1). Ossification of the thyroid cartilage was observed in all of the participants. Additionally, 26 patients underwent selective unilateral (n = 5) or bilateral (n = 21) neck dissection at levels II–IV. In total, 13 patients underwent postoperative radiotherapy, including 2 cases whose surgical margins were positive. The tumors were found to extend to the thyroid cartilage in 12 T3 and T4 cases, and 8 of these cases exhibited involvement of the intermediate lamina of the thyroid cartilage (thyroid cartilage at the AVC region, ITC).

**TABLE 1:**
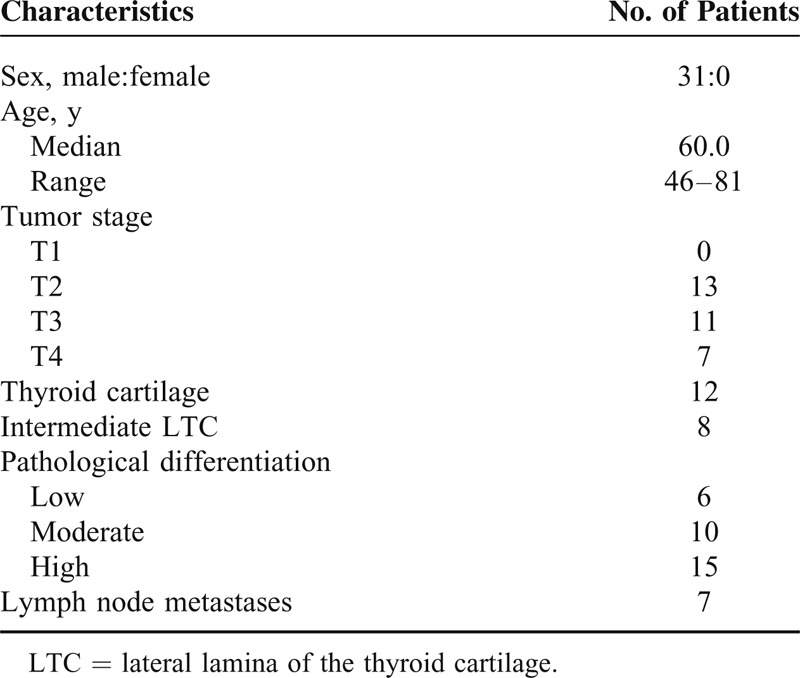
Patient Characteristics

### Tumor Extension

The glottic carcinomas were found to extend into the supraglottic region in 27 patients (87.1%), and this extension was via the AVC in only 4 (4/27, 14.8%) cases. In the other 20 cases (20/27, 85.2%), the tumors extended either through the paraglottic space or along the surface of the vocal cord out of the AVC and resulted in the involvement of Galen's ventricles and/or the ventricular folds. The subglottic region involvement was observed in 21 patients (67.7%), and the tumors extended via the AVC in 15 (15/21, 71.4%) of these cases. The subglottic region was more frequently involved than the supraglottic region via the AVC (71.4% vs 14.8%).

### Involvement of Broyles’ Ligament and the Vocal Cords

#### Involvement of the Vocal Cords

**FIGURE 1:**
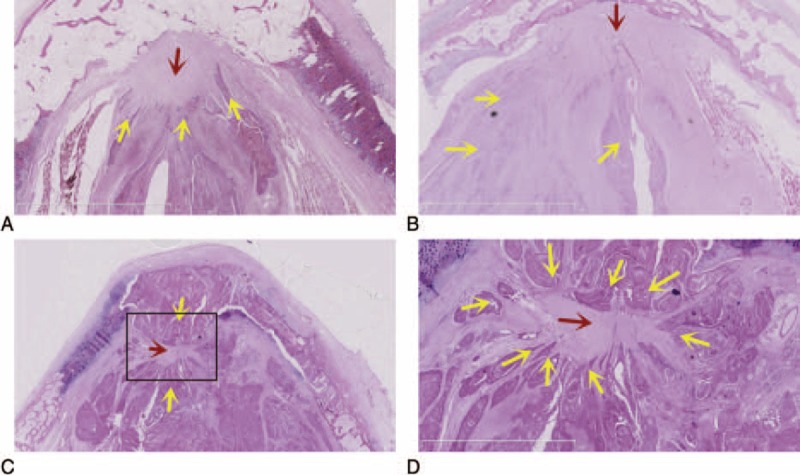
Involvement of the vocal cords and Broyles’ ligaments. (A) Involvement of the superficial layers of the bilateral vocal cords in an AVC2 case without involvement of the vocal muscle in the AVC region. (B) Involvement of the superficial layers of the unilateral vocal cords or deep layers of the contralateral in an AVC2 case with involvement of the vocal muscle in the AVC region. (C) The involvement of the deep layers of the bilateral vocal cords in an AVC3 case with involvement of the vocal muscle and the ITC in the AVC region. (D) Magnified image of the areas in the rectangle in (C), ×2.5; laryngeal tumors “ringing” the Broyles’ ligaments and not breaking through to the ventral and dorsal ligaments. H&E, yellow arrows: tumor cells; red arrows: Broyles’ ligament; black arrows: thyroid cartilage. AVC = anterior vocal commissure, H&E = hematoxylin–eosin staining.

The involvement of the superficial layers (epithelial layers or propria lamina) of the unilateral or bilateral vocal cords was present in 15 cases (48.4%; Figure 1A) without the involvement of vocal muscle in the AVC region; of these cases, 4 were AVC1 and 11 were AVC2.

The involvement of the superficial layer of the unilateral vocal cord and the deep layer (vocal muscle or deeper structures) contralaterally occurred in 9 cases (29.0%; Figure 1B) of which 3 were AVC2 without the involvement of the vocal muscle in the AVC region (WVM) and 6 were AVC2 with the IVM.

The involvement of the deep layers of the bilateral vocal cords occurred in 7 cases (22.6%; Figure 1C); all of these cases included involvement of the vocal muscle in the AVC and were classified as AVC3.

#### Involvement of Broyles’ Ligament

Based on the vocal muscle of the AVC region, we defined 2 groups. The first group was WVM. In 18 patients (58.1%), the laryngeal tumor invaded only into the dorsal side (epithelial layers) of Broyles’ ligament (Figure 1A).

The second group exhibited IVM. In 13 cases (41.9%), the laryngeal tumors were observed to “ring” the Broyles’ ligaments to varying degrees and did not break through the ventral and dorsal ligaments (cartilage to mucosa) in any case (Figure 1D).

### Involvement of ITC and Its Relation With Broyles’ Ligament

**FIGURE 2:**
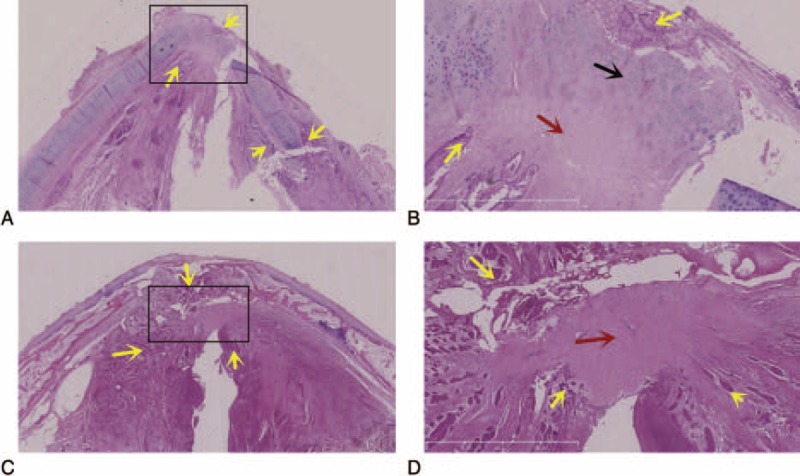
Involvement of the ITC. (A, B) Amplified image of the rectangle in (A), ×2.5; the ITC outer plate presented with minifocal destruction in a case with unilateral involvement of the LTC. (C, D) Amplified image of the rectangle in (C), ×2.5; the ITCs exhibited bulk destruction of the inner and outer plates with bilateral involvement of the LTC. H&E, yellow arrows: tumor cells; red arrows: Broyles’ ligament; black arrows: thyroid cartilage). H&E = hematoxylin–eosin staining, ITC = intermediate lamina of the thyroid cartilage, LTC = lateral lamina of the thyroid cartilage.

In total 8 cases (25.9%), the involvement of the ITC was observed. In 2 of these 8 patients, the ITC exhibited minifocal destruction and the unilateral involvement of the lateral lamina of the thyroid cartilage (LTC; Figure 2A and B). In the remaining 6 cases, the ITC exhibited bulk destruction of the inner and outer plates and the bilateral involvement of the LTC (Figure 2C and D). In the WVM and IVM groups, involvement of the ITC occurred in 0.0% and 53.5% (0/18 and 8/13) of the cases, respectively (*P* < 0.05).

### Surface Morphology and Tumor Growth Pattern

**FIGURE 3:**
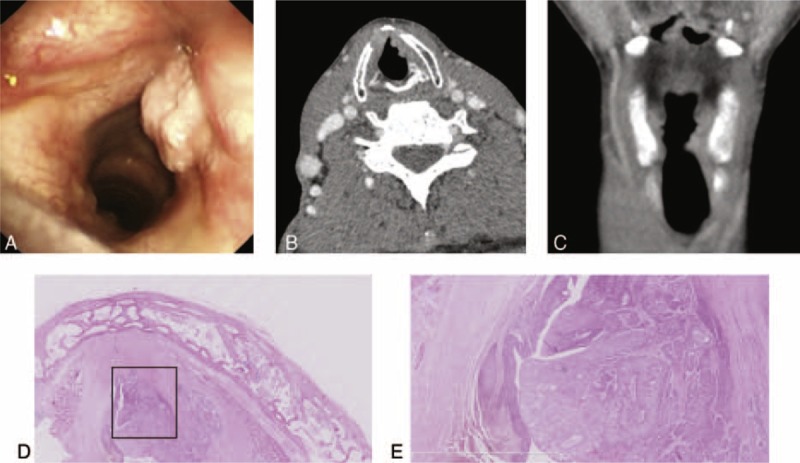
Pushing carcinoma. (A) Electronic laryngoscopic, (B) CT, axial, and (C) CT, coronal. A tumor with its main body located in the larynx cavity with a clear tumor edge surface morphology. (D, E) H&E, amplified image of the rectangle in (D), ×2.5. The tumor exhibited well-defined tumor mass. CT = computed tomography, H&E = hematoxylin–eosin staining.

**FIGURE 4:**
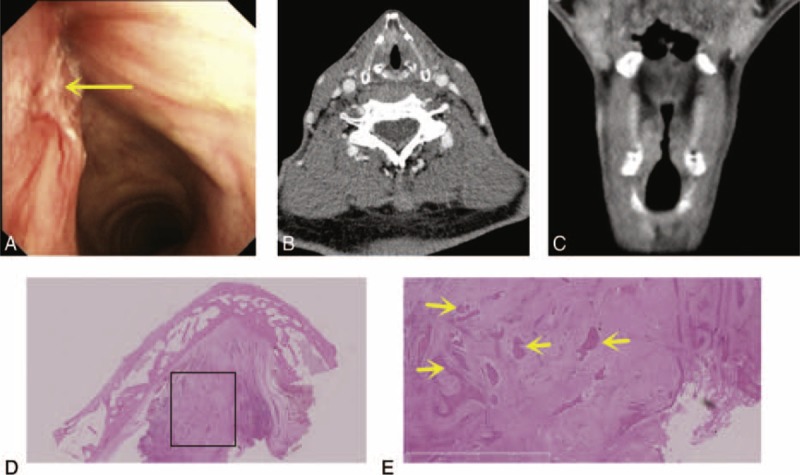
Infiltrating carcinoma. (A) Electronic laryngoscopic, (B) CT, axial, and (C) CT, coronal. A tumor with its main body in the vocal propria lamina and the deeper layers and exhibiting an unclear tumor edge surface morphology. (D, E) H&E, amplified image of the rectangle in (D), ×2.5. This tumor exhibited multiple tumor nests that were often surrounded by fibrosis (yellow arrows: tumor cells or tissue). CT = computed tomography, H&E = hematoxylin–eosin staining.

In the pushing carcinomas (Figure 3) and the infiltrating carcinomas (Figure 4), 15 of 21 (71.4%) and 2 of 10 (20.0%), respectively, of the cases presented with well-defined tumor mass (*P* < 0.05). Eight infiltrating carcinomas presented with multiple tumor nests that were often surrounded by fibrosis (Figure 4E).

## DISCUSSION

At present, there is no consensus about the contribution of the AVC and its related structures to the infiltration of laryngeal carcinomas.^4^ Compared with the without AVC involvement, patients with laryngeal carcinomas that compromises the AVC suffer from a higher recurrence rate,^2^ which may be due to the determination of the tumor extension and therapeutic strategy decisions.

Imaging scan occasionally miss or overestimate the tumor extension and the involvement of the thyroid cartilage.^14^ In our study, we attempted to reveal a novel signature and approach of laryngeal cancer that infiltrates the AVC with histopathological examinations to identify additional features and possible invasion paths of glottic carcinomas with AVC involvement.

### Tumor Extension

In our study, the glottic carcinomas were found to extend into the supraglottic region in 27 patients (87.1%), and only 4 (4/27, 14.8%) of these carcinomas extended via the AVC. The involvement of the subglottic region was observed in 21 patients (67.7%), and in 15 (15/21,71.4%) of these cases, the tumor extended via the AVC. Some studies have indicated that although the thyroepiglottic ligament in the supraglottis and Broyles’ ligament in the glottis region share similar components, the thyroepiglottic ligament is rich in glands and vessels that are absent in Broyles’ ligament.^15^ There is a separate region termed the “0-plane” between the thyroepiglottic ligament and Broyles’ ligament; this region lacks glands and vessels and is thus believed to be capable of effectively stopping tumor infiltration.^16,17^ However, glands and vessels are continuously present in the glottic and subglottic regions.^18^ Similar to other reports, we found that the involvement of the subglottic region via the AVC was more frequent than the involvement of the supraglottic region via the AVC (71.4% vs 14.8%, respectively). Therefore, to some extent, the AVC functions as a barrier that prevents glottic tumors from breaking into the supraglottic region, but the AVC also acts as a pathway for the spread of cancer downward to the subglottis.

### Involvement of the ITC and Broyles’ Ligament

In our study, 18 patients (58.1%) exhibited laryngeal tumors that invaded only the dorsal side (epithelial layers) of Broyles’ ligaments and did not involve the ITC (Figure 1A), suggesting that Broyles’ ligament did act as a barrier against tumor invasion to the ITC to some extent. This barrier function may be because of the compact connective tissue and the absence of glands and vessels; these characteristics inhibit infiltration.^13^

In the 8 cases of ITCs, the ITCs exhibited minifocal destruction, and unilateral involvement of the LTC was present in 2 cases (Figure 2A and B). In the remaining 6 cases, the ITC exhibited bulk destruction of the inner and outer plates with bilateral involvement of the LTC (Figure 2C and D). However, all of these 8 exhibited laryngeal tumors that “ringed” the Broyles’ ligaments to varying degrees and did not break through to the ventral or dorsal ligaments. This pattern indicates that Broyles’ ligament did act indirectly as a barrier against the spread of the tumors to the thyroid cartilage to some extent.

In the WVM and IVM groups, the rates of the involvement of the ITC were 0.0% and 53.5% (0/18 vs 8/13; *P* < 0.05), respectively. When the involvement of the vocal muscle of the AVC region, the involvement of the ITC was not found in the WVM group, whereas the involvement of the ITC was obviously more frequent in the IVM group (8/13). These findings indicate that Broyles’ ligament acted as a barrier against the spread of the tumor to the thyroid cartilage, but this function as a barrier was obviously weakened by the IVM.

The vocal muscles might provide a potential path for a tumor to bypass Broyles’ ligament and then compromising the thyroid cartilage. The vocal muscles that insert into Broyles’ ligament are extremely close to the ITC. The tumoral involvement of the cartilage is thought to be promoted by the chemotactic effect of vascular endothelial growth factor.^19^ The ossification of the thyroid cartilage is another reason for cartilage involvement. The ossification of the ITC occurs in front of the LTC due to the insertion, the pulling from the vocal muscles, and the cartilage angle.^20,21^ The ossification of thyroid cartilage typically begins when people are approximately 30 years old and forms an abundant vascular system that facilitates tumoral invasion.^20^ Compared with ossific cartilage, hyaline cartilage exhibits greater resistance to tumors because of its compact structure and deficiency of vessels.^21^ In our study, ossification of the observed ITC was found in all the 31 cases.

### Surface Morphology and Tumor Growth Pattern

The pushing carcinomas (Figure 3) and the infiltrating carcinomas (Figure 4) exhibited tumor growth patterns on the preoperative electronic laryngoscopic examinations and CT scans (*P* < 0.05). Fifteen of the 21 (71.4%) pushing tumors presented with well-defined tumor mass. Eight infiltrating carcinomas presented with multiple tumor nests that were often surrounded by fibrosis. Some authors have reported that the growth patterns of recurrent carcinomas and primary carcinomas are different and that most recurrent carcinomas present with multiple tumor nests.^22^

### Choice of Transoral CO_2_ Laser Microsurgery

Many authors have attempted to apply transoral CO_2_ laser microsurgery to treat early glottic carcinomas with AVC involvement,^23^ but this technique remains controversial and the indications for this treatment are not unified.^12,24^

In our study, the Broyles’ ligament involvement also drew our attention because no tumors penetrated across the ventral and dorsal aspects of the ligament in any case in our study. Thus, this ligament can act as a barrier to protect the thyroid cartilage from tumoral invasion at least in early cases, and this role is similar to that of the perichondrium. Therefore, transoral CO_2_ laser microsurgery is not contraindicated for early glottic carcinomas with AVC involvement. Regarding the involvement of the vocal superficial layer and the AVC1 and AVC2 categories, which do not exhibit thyroid cartilage involvement, we recommend the use of transoral CO_2_ laser surgery. Laser microsurgery ensures complete tumor resection and an adequate safety margin by cutting the tissue between Broyles’ ligament and the ITC. However, regarding the involvement of superficial vocal layer, AVC3 category, and the involvement of the deep vocal layer in AVC1 or AVC2 cases, caution should be exercised regarding the use of transoral CO_2_ laser microsurgery. The clinical T stage, degree of AVC involvement, thyroid cartilage involvement, vocal cord movement, and tumor exposure need to be assessed. To improve tumor exposure in cases of difficult exposure, we recommend the use of laryngoscopy and rigid endoscopes (30°, 45°, and 70°) to watch the lesion sites. We should also note that cases with vocal activity limitations should not be considered for laser surgery. Hartl et al^25^ studied 94 cases with the tumors that involved the AVC and suggested that vocal fold mobility was the only factor that was significantly related to thyroid cartilage invasion. Regarding the involvement of the deep layers of the bilateral vocal cords and AVC3 cases, we advise that transoral CO_2_ laser microsurgery not be used because the ITC may be involved.

We also need to focus on tumor growth patterns in relation to the use of CO_2_ laser microsurgery. Infiltrating carcinomas present with multiple tumor nests that are often surrounded by fibrosis and are often understaged.^22^ Therefore, preoperative electronic laryngoscopic examinations and CT scans should be used to assist the determination of the tumor growth pattern.
